# SrTiO_3_ Cubes and Truncated Rhombic Dodecahedra Exhibiting Large Lattice and Piezoelectric Variations

**DOI:** 10.1021/acs.jpclett.5c01107

**Published:** 2025-05-10

**Authors:** Bo-Hao Chen, Satyaranjan Jena, Ya-Ju Chuang, Hsun-Yen Lin, Chih-Hsueh Li, Jyh Ming Wu, Michael H. Huang

**Affiliations:** † Department of Chemistry, 34881 National Tsing Hua University, Hsinchu 300044, Taiwan; ‡ 57815 National Synchrotron Radiation Research Center, Hsinchu 300092, Taiwan; § Department of Materials Science and Engineering and High Entropy Materials Center, 34881 National Tsing Hua University, Hsinchu 300044, Taiwan

## Abstract

Recognizing the existence of notable X-ray diffraction (XRD) peak shifts between SrTiO_3_ cubes and {100}-truncated rhombic dodecahedra, their synchrotron XRD patterns were analyzed. Again significant peak shifts are present. Moreover, cubes give symmetric diffraction peaks, yet truncated rhombic dodecahedra exhibit peak splitting that can be deconvoluted into bulk and (110)- and (110)-related surface lattice components. Temperature-varying XRD measurements reveal different cell constant expansion and contraction behaviors for these two samples. Fast Fourier transform (FFT) processing of their high-resolution transmission electron microscopy (HR-TEM) images indicate a thinner surface layer lattice with considerable lattice point deviations for a truncated rhombic dodecahedron than that for a cube, explaining the observation of XRD peak splitting. The truncated rhombic dodecahedra also show much larger piezoelectric and ferroelectric responses than the cubes do. The diverse facet-dependent behaviors of SrTiO_3_ crystals are linked to their interior lattice variations.

## Graphical abstract

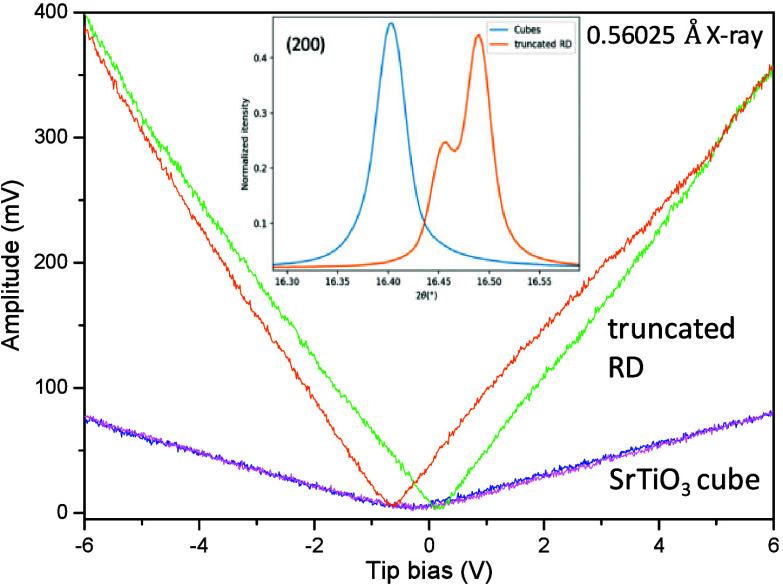

Semiconductor crystals generally exhibit facet-dependent electrical conductivity, photocatalytic activity, and optical properties.^1–10^ For magnetic and piezoelectric materials, such as MnS, Co_3_O_4_, BaTiO_3_, and PbZrTiO_3_ nanocrystals, their magnetic and piezoelectric responses also display shape dependence.^11–14^ All these phenomena are related, and they can be understood to arise from the presence of a surface layer inside a crystal with facet-specific lattice variations, just like a cubic piece of meat with the opposite faces heated to different extents can produce dissimilar flavors, depending on the cut of the meat.^15^ After early density functional theory (DFT) predictions, synchrotron XRD patterns and fast Fourier transform (FFT) conversion of HR-TEM images reveal the presence of bulk and surface layer lattices in Cu_2_O and other crystals.^16–19^ Consequently, dielectric constant values can also change for different particle shapes.^13,14^ The facet-related lattice deviations should affect charge transfer across a specific surface and absorb somewhat different light wavelengths. Strontium titanate is another material showing facet-dependent photocatalytic activity, electrical conductivity, and optical properties.^20–23^ The {110} faces of SrTiO_3_ are much more electrically conductive than its {100} faces, and the {100}-truncated rhombic dodecahedra are much more photocatalytically active than the cubes.^20,21^ Similarly sized SrTiO_3_ cubes also show a larger band gap than that of the truncated rhombic dodecahedra.^20^ It has a perovskite crystal structure with a direct band gap of 3.75 eV and an indirect band gap of 3.25 eV.^24^ Its band energy positions are similar to those of TiO_2_ for possible photocatalytic water splitting application.^25^ Previously, in-house XRD characterization on SrTiO_3_ cubes, edge-truncated cubes, and {100}-truncated rhombic dodecahedra has revealed notable diffraction peak shifts among these samples.^20^ Thus, their lattice constants differ considerably. TEM examination also confirmed changes in their fringe spacing. However, such XRD peak shifts often get ignored, so facet-dependent photocatalytic behaviors are generally explained in terms of surface atom arrangement.^26,27^ The surface atom arrangement or geometry is unable to explain why the same semiconductor material can exhibit diverse colors. The large XRD peak shifts for different SrTiO_3_ particle shapes suggest that further structural analysis through synchrotron XRD patterns and FFT-processed HR-TEM images is necessary.

Here, SrTiO_3_ cubes and {100}-truncated rhombic dodecahedra have been prepared again for synchrotron XRD characterization, showing very different peak features and large peak shifts. Temperature-varying XRD patterns also present notable differences. Their FFT-converted HR-TEM images reveal greater lattice point deviations in the cube sample. These lattice features should give rise to the exhibited property changes. For further property characterization of these particles to demonstrate lattice-induced effect, their piezoelectric and ferroelectric responses were recorded, showing that notable differences still exist.

The 160-nm SrTiO_3_ cubes and {100}-truncated rhombic dodecahedra with an opposite face length of 168 nm were synthesized following the reported conditions.^20^ In addition to different solvents added, the reagent amount and the reaction temperature were changed to produce these particle shapes. These changes mean that the reaction quotient *Q* is different in making the cubes and truncated rhombic dodecahedra. Consequently, the reaction free energy change Δ*G* can vary, since Δ*G* = Δ*G°* + *RT* ln *Q*. While Δ*H* in the crystal growth process is not known, it is possible that the reaction entropy change (Δ*S*) is not the same for these particles, as Δ*G* = Δ*H* – *T*Δ*S*. The lattice entropy can be directly observed, especially after FFT processing of the collected HR-TEM images. This analysis demonstrates that shape-related lattice deviations occur naturally as required by thermodynamics.

Figure 1 shows SEM images of the prepared SrTiO_3_ cubes and {100}-truncated rhombic dodecahedra. The particles have fairly high size and shape uniformity. XRD patterns of the SrTiO_3_ cubes and truncated rhombic dodecahedra using an in-house X-ray diffractometer are also provided in Figure 1. Significant peak shifts are identified, with the strongest (110) peak located at 32.32° and 32.49° 2θ, respectively. However, the peak shifts cannot be easily recognized when looking at the whole XRD patterns. This is why peak shifts are often overlooked. To reveal more details of the peak shifts, synchrotron XRD patterns were collected (Figure S1). By overlapping XRD patterns of SrTiO_3_ cubes and truncated rhombic dodecahedra, peak shifts are easily seen. The degree of shifts becomes larger at higher 2θ angles. Clear peak shifts are observed when expanding selected (110), (111), and (200) peaks. More interestingly, peak splitting is present for the truncated rhombic dodecahedra. Rietveld refinement was then carried out to fit the diffraction peaks into bulk and surface layer components (Figure S2). As seen in Figure 2, the peak symmetry means that the cubes can only be fitted using a single component. Interestingly, the splitted peak feature of the truncated rhombic dodecahedra can be deconvoluted into three components, representing the interior bulk and the surface layer regions related to the (110) and (100) planes or faces. The (110) component is largely responsible for the peak splitting. The results demonstrate substantial lattice differences between the two particle shapes. Table S1 summarizes the Rietveld refinement results. The peak components with large microstrains are designated as the surface lattices, since the crystal surface is more likely to experience lattice strain. The cubes have a cell constant *a* of 3.927 Å. For the truncated rhombic dodecahedra, the bulk cell constant is 3.907 Å, while the {110}- and {100}-related surface components have cell constants of 3.916 and 3.911 Å, respectively. Please note that a cell constant difference of 0.021 Å is large enough to be easily recognized by the synchrotron X-ray facility and even an in-house X-ray diffractometer. Figure S3 shows the surface layer can reach 9 nm into the crystal interior, as determined using the weight percentages of the surface layer and bulk components.

**Figure 1:**
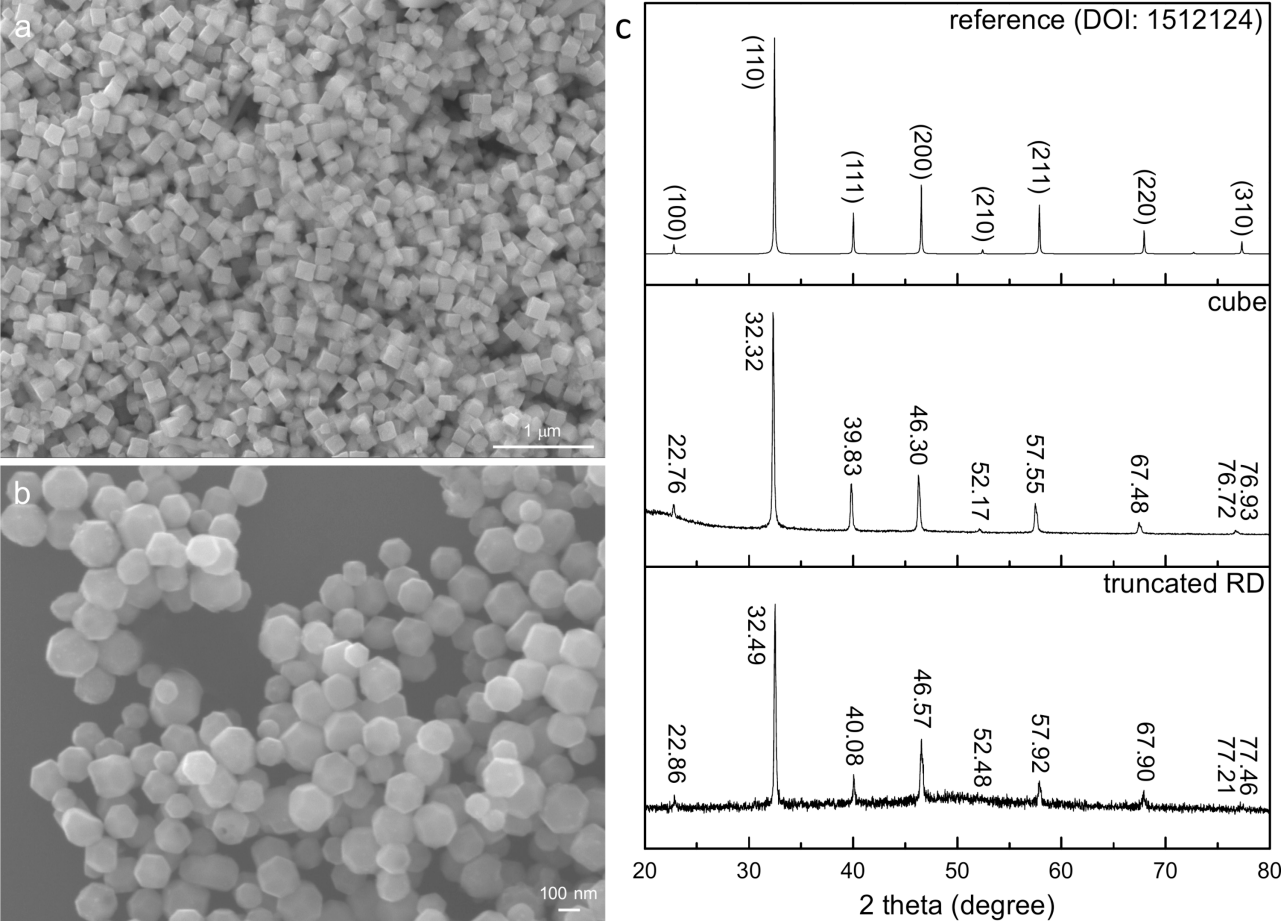
SEM images of the synthesized SrTiO_3_ (a) cubes and (b) truncated rhombic dodecahedra. (c) XRD patterns of SrTiO_3_ cubes and truncated rhombic dodecahedra. A reference pattern is provided.

**Figure 2:**
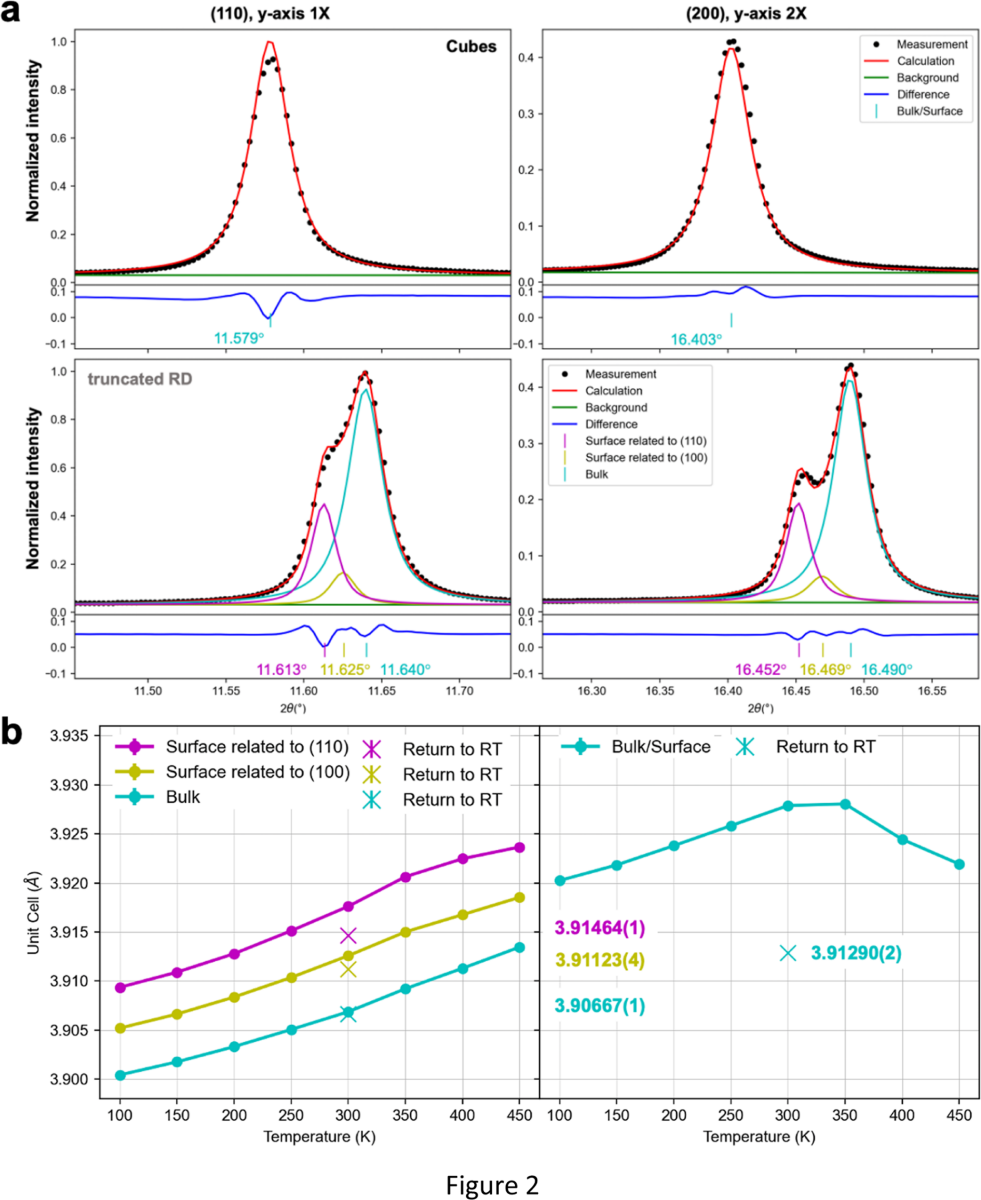
(a) XRD patterns of SrTiO_3_ cubes and truncated rhombic dodecahedra after the Rietveld refinement. (b) Unit-cell variation of SrTiO_3_ truncated rhombic dodecahedra and cubes at different heating temperatures and after returning to room temperature. Unit-cell constants of bulk and surface layer regions after returning to room temperature are shown.

Since the lattice constant variations are related to the crystal entropy, it is interesting to subject the crystals to low and high temperatures to see how lattice constants are varied. SrTiO_3_ cubes and truncated rhombic dodecahedra were first cooled to 100 K for the XRD measurements, and the sample temperature was raised by 50 K to collect the next diffraction patterns. The samples were eventually heated to 450 K, and allowed to return to room temperature for the final measurements. Figure S4 shows the strongest (110) peak shifts of the two samples, while Figure 2b summarizes the changes in the unit-cell constant. With increasing temperature, the bulk and surface layer lattices expand continuously as expected. The unit-cell constant increases by about 0.013 Å from 100 K to 450 K for the truncated rhombic dodecahedra. By comparison, the 0.021 Å cell constant difference between cubes and truncated rhombic dodecahedra is enormous. Upon returning to room temperature, the bulk cell constant shrinks to the same length as that recorded at 300 K. However, while the cell constants in the surface layer region also shrink, their lengths are slightly shorter than when the truncated rhombic dodecahedra first reached 300 K. This means that the heated particles are not identical to their preheated state. Ag_2_O crystals show a similar behavior.^19^ Just like cooking a piece of meat, the meat is not the same after heat is removed as it was before heat was applied. The large lattice distortion or stress generated in ionic solids during heating may not be completely released after cooling. This is the lattice origin for the emergence of facet-dependent behaviors. By contrast, the unit-cell constant of SrTiO_3_ cubes increases at temperatures up to 300–350 K, followed by a large lattice shrinkage with further heating to 450 K. Upon cooling the cubes to room temperature, a huge lattice shrinkage was recorded. There is a lattice shrinkage of 0.015 Å between the unheated and heated cubes. Obviously, the two samples behave quite differently to result in facet-dependent properties.

TEM and HR-TEM images of a SrTiO_3_ cube are shown in Figure 3. FFT-processed lattice point images are also presented. The selected-area electron diffraction (SAED) pattern indicates the particle is a single crystal with (200) set of spots pointing toward the cubic faces. Lattice fringes indicate the (110) planes are aligned toward the cube edge, while the (200) planes are oriented parallel to the cube face. More importantly, the lattice point images reveal significant deviations in the appearance of the spots near the surface layer region, or around 5 nm within the crystal surface (see Figures 3d and 3g). The spots have become elongated and point along certain directions. This is the visual evidence of the surface layer with lattice disturbance that extends beyond 5 nm. By contrast, the interior lattice spots have mostly a rounded dot shape as normally expected. Figure 4 gives the same HR-TEM characterization on a {100}-truncated rhombic dodecahedron. The (200) set of spots also orient along the {100} faces of the particle. The FFT-processed lattice point images are shown (see Figures 4d–g). The lattice points within 2 nm into the surface have far less deviations, with elliptical spots pointing along the (110) plane direction. However, beyond 2 nm into the crystal interior, the separated spots can begin to stretch and link together, or become elongated and point around 45°, with respect to the (110) and (200) planes (Figure 4d). The observations agree with the synchrotron XRD differences between the two samples, that the truncated rhombic dodecahedra possess interior and surface layer lattices. For the cubes, the thick surface layer means that it appears more homogeneous to X-ray diffraction to give symmetric peaks. TEM analysis indicates that the surface layer thickness is critical to the appearance of single or splitted diffraction peaks. MnS crystals show similar symmetric XRD peaks from the presence of a thick surface layer having considerable lattice deviations.^11^

**Figure 3:**
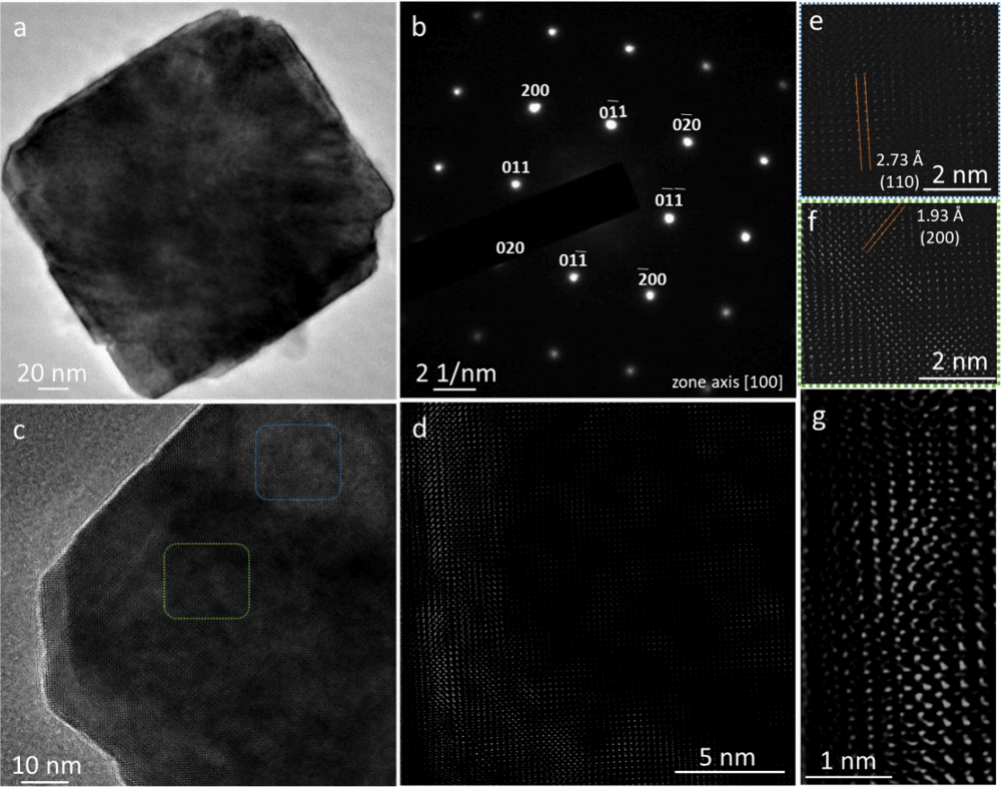
(a, b) TEM image of a SrTiO_3_ cube and its corresponding SAED pattern. (c) HR-TEM image of a corner of another cube. (d) FFT-processed HR-TEM image of the surface region in panel (c). (e, f) FFT-processed lattice point image corresponding to the blue and green square regions in panel (c). (g) Magnified image of the edge portion in panel (d).

**Figure 4:**
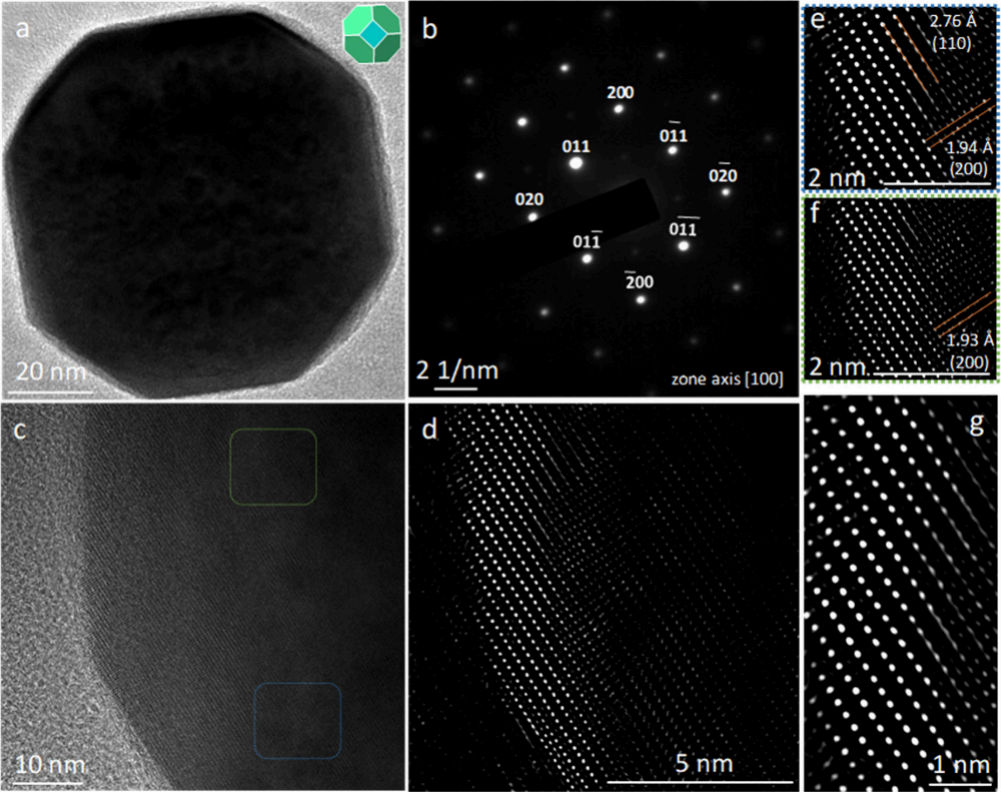
(a, b) TEM image of a SrTiO_3_ truncated rhombic dodecahedron, its drawing, and the corresponding SAED pattern. (c) HR-TEM image of another truncated rhombic dodecahedron. (d) FFT-processed HR-TEM image of panel (c). (e, f) FFT-processed lattice point image corresponding to the blue and green square regions in panel (c). (g) Magnified image of the edge portion in panel (d).

SrTiO_3_ is a ferroelectric material that can be applied in information storage.^28^ To demonstrate how the interior lattice variations affect the physical properties of these crystals, their piezoelectric curves and ferroelectric loops were obtained through the use of piezoresponse force microscopy. Figure 5 gives the measured piezoelectric butterfly curves and ferroelectric hysteresis loops of the SrTiO_3_ cubes and truncated rhombic dodecahedra. The truncated rhombic dodecahedra have much higher piezoelectric voltage generation than that of the cubes, and they also present bigger ferroelectric hysteresis loops. Because piezoelectricity results from the alignment of electric dipoles of ions in a crystal under an applied electric field, lattice variations between a SrTiO_3_ cube and a truncated rhombic dodecahedron could yield dissimilar degrees of dipole alignment to result in different piezoelectric responses. Clearly, the interior lattice variations induce these intrinsic property changes.

**Figure 5:**
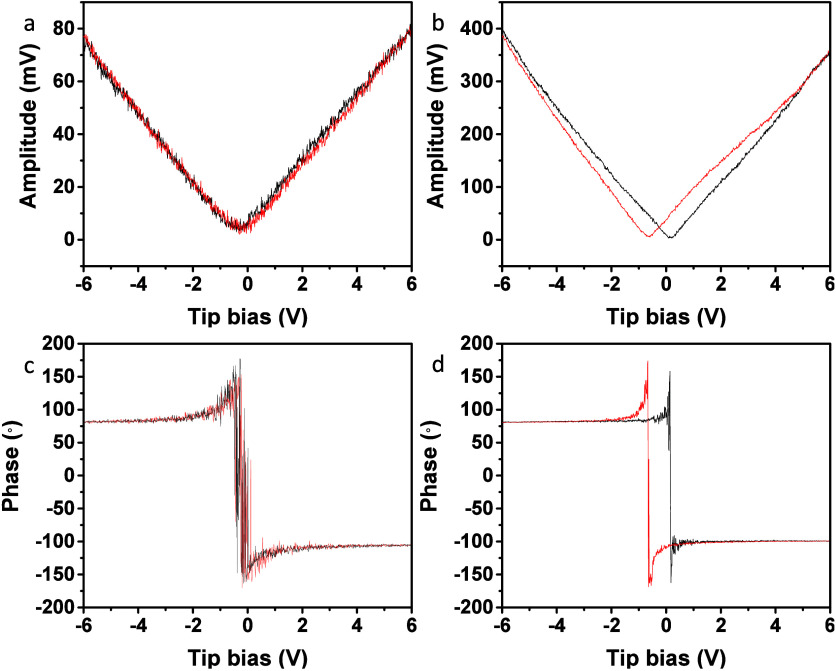
Piezoelectric curves and ferroelectric hysteresis loops of a SrTiO_3_ (a, c) cube and (b, d) a truncated rhombic dodecahedron.

In conclusion, SrTiO_3_ cubes and {100}-truncated rhombic dodecahedra were prepared for synchrotron XRD analysis. While cubes have symmetric peaks, truncated rhombic dodecahedra show peak splitting, in addition to their large differences in the unit-cell constant. The peak splitting indicates the presence of bulk and surface layer lattices. While truncated rhombic dodecahedra show continuous lattice expansion from 100 K to 450 K, cubes reach a maximum cell constant around 300–350 K, followed by a decreased cell constant at higher temperatures. FFT-processed HR-TEM lattice point images reveal the surface layer of cubes is notably thicker than that of the truncated rhombic dodecahedra, explaining why symmetric XRD peaks are observed for the cubes. The truncated rhombic dodecahedra exhibit considerably larger piezoelectric and ferroelectric responses than the cubes do. The interior lattice deviations produce various facet-dependent phenomena.

## Supplementary Material



## Notes

XRD patterns, Rietveld refinement results, and a model of truncated rhombic dodecahedra showing the surface layer thickness (PDF)
