# Supplementary material for: SrTiO3 Cubes and Truncated Rhombic Dodecahedra Exhibiting Large Lattice and Piezoelectric Variations
Source: J Phys Chem Lett. 2025 May 10;16(20):4921–6. doi: 10.1021/acs.jpclett.5c01107 (PMC12104999; doi:10.1021/acs.jpclett.5c01107)
Supplement: Supplementary file 1 [file jz5c01107_si_001.pdf]

Supporting Information

$SrTiO_3$ Cubes and Truncated Rhombic Dodecahedra Exhibiting Large Lattice and Piezoelectric Variations

Bo-Hao Chen,[†,‡] Satyaranjan Jena,[†] Ya-Ju Chuang,[†] Hsun-Yen Lin,[§] Chih-Hsueh Li,[†] Jyh Ming Wu,[§] and Michael H. Huang*,[†]
[†]Department of Chemistry, National Tsing Hua University, Hsinchu 300044, Taiwan
[‡]National Synchrotron Radiation Research Center, Hsinchu 300092, Taiwan
[§]Department of Materials Science and Engineering and High Entropy Materials Center, National Tsing Hua University, Hsinchu 300044, Taiwan
Email: hyhuang@mx.nthu.edu.tw

EXPERIMENTAL SECTION

Chemicals. Strontium chloride hexahydrate ($SrCl_2{\cdot}6H_2O$, 99%, J. T. Baker), titanium tetrachloride ($TiCl_4$, 98%, SHOWA), lithium hydroxide monohydrate ($LiOH{\cdot}H_2O$, 98%, Alfa Aesar), ethanol (C_2H_5OH, ≥99.5%, Honeywell), and ethylene glycol ($C_2H_6O_2$, 99+%, ACROS ORGANICS) were used as received.

Synthesis of $SrTiO_3$ Cubes. First, 0.031 mL of $TiCl_4$ was added to 2.5 mL of ethanol and stirred for 10 min. Next, 1 mL of an aqueous solution containing 0.084 g of $SrCl_2{\cdot}6H_2O$ was added and stirred for 5 min, followed by the introduction of 3.7 mL of 3 M LiOH aqueous solution with stirring for 30 min. The solution was transferred to a Teflon container and heated at 70 ºC for 3 h. The resulting precipitate was collected under 10000 rpm centrifugation for 4 min, followed by sonication with ethanol and deionized water three times each.

Synthesis of $SrTiO_3$ Truncated Rhombic Dodecahedra. First, 0.026 mL of $TiCl_4$ was added to a mixed solution of 2 mL of water and 0.85 mL of ethylene glycol and stirred for 5 min. Next, 1 mL of an aqueous solution containing 0.070 g of $SrCl_2{\cdot}6H_2O$ was added and stirred for 10 min, followed by the introduction of 3.7 mL of 3 M LiOH aqueous solution with stirring for 30 min. The solution was transferred to a Teflon container and heated at 200 ºC for 20 h. The resulting precipitate was collected under 10000 rpm centrifugation for 4 min, followed by sonication with ethanol and deionized water three times each.

Piezoelectric and Ferroelectric Response Measurements. Piezoresponse force microscopy (PFM, Bruker Dimension ICON) was employed to investigate the high-speed piezoelectric response, butterfly curves, and hysteresis loops of the samples. In general, a suitable quantity of the material is dispersed in absolute ethanol and subjected to ultrasonic dispersion for 30 min. Following this, the well-dispersed solution is carefully deposited onto an indium tin oxide substrate and subsequently dried in an oven to ensure uniform film formation.

Instrumentation. A thermal field emission scanning electron microscope (JEOL JSM-70000F) was used. XRD patterns were taken using a Bruker D2 Phaser powder X-ray diffractometer with Cu *K*α radiation. Synchrotron XRD patterns were collected at the Taiwan Photon Source 19A beamline (TPS19A). The hard X-ray source was delivered from an in-vacuum cryogenic undulator (CU15), and the diffraction patterns were recorded using a position-sensitive detector MYTHEN 18K. TEM characterization was performed using a JEOL ARM-200F electron microscopes with an accelerating voltage of 200 kV.

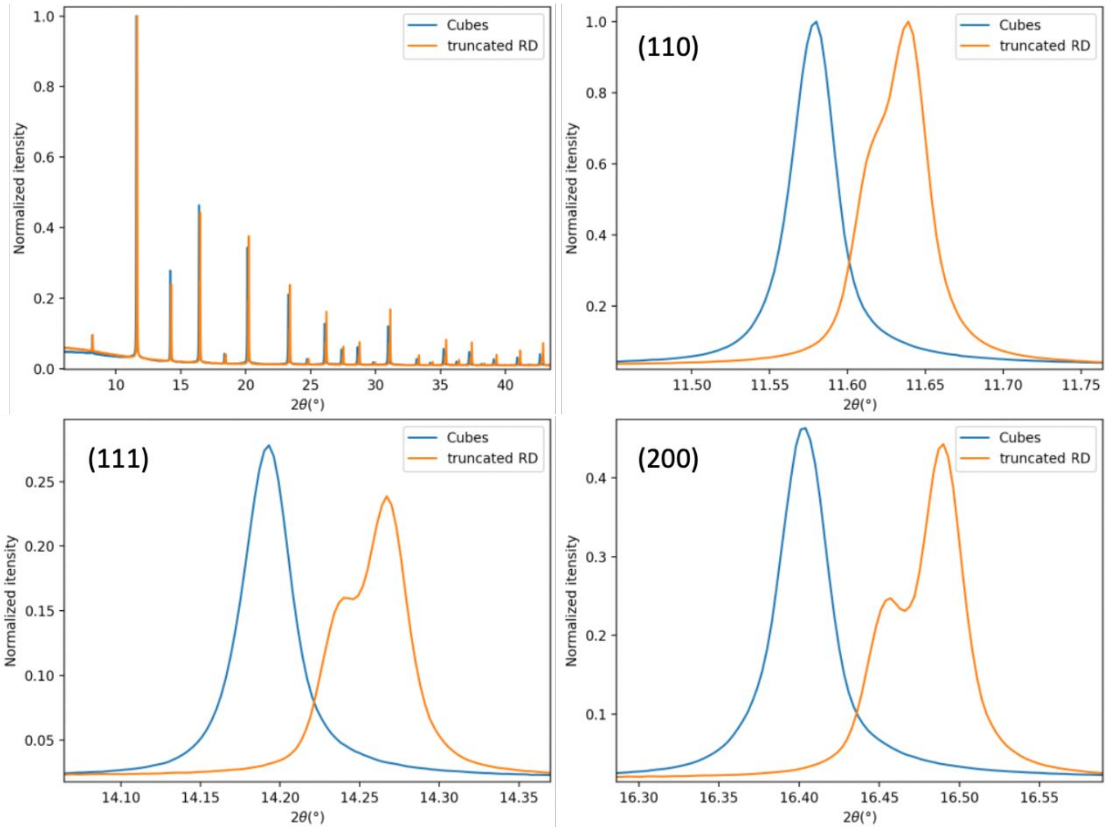

Figure S1. Synchrotron XRD patterns of the synthesized $SrTiO_3$ cubes and truncated rhombic dodecahedra. Selected peaks are highlighted.

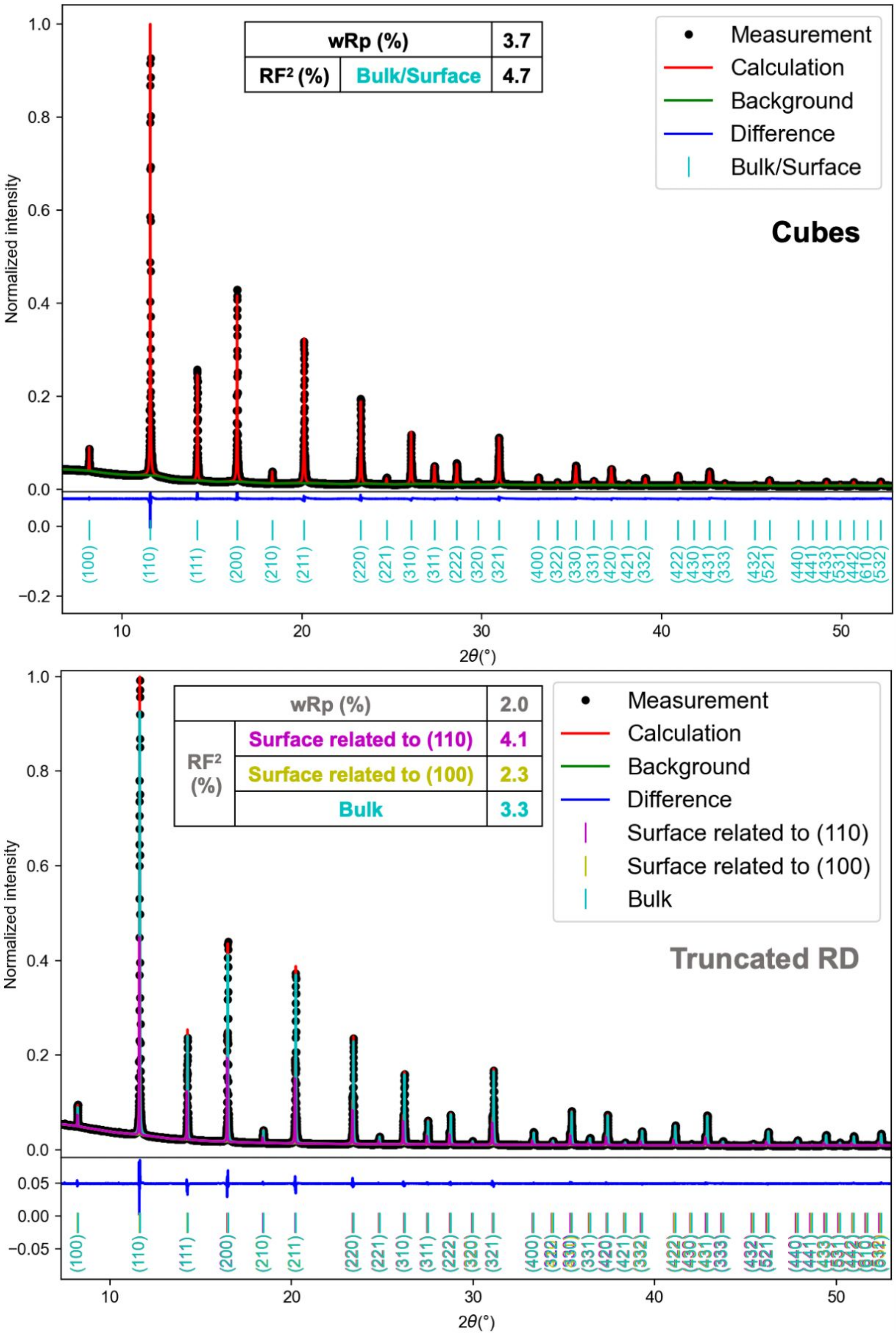

Figure S2. Rietveld refinement of the collected XRD patterns of $SrTiO_3$ crystals.

Table S1. Rietveld Refinement Results for $SrTiO_3$ Cubes and {100}-Truncated Rhombic Dodecahedra

Morphology	**Cubes**	**Truncated Rhombic Dodecahedra**		
		**Surface (110)**	**Surface (100)**	**Bulk**
a (Å)	3.92741(2)	3.91564(2)	3.91151(5)	3.90669(1)
**Δcell (Å)**	**0.02072(2)**	**0.00895(2)**	**0.00482(5)**	**-**
Return to RT, a (Å)	3.91290(2)	3.91464(1)	3.91123(4)	3.90667(1)
Return to RT, **Δcell (Å)**	**0.00623(2)**	**0.00797(1)**	**0.00456(4)**	**-**

Formular	$SrTiO_3$			
FW(g/mol)	183.52			
Space group	*P* m-3 m (223)			
λ (Å)	0.56025			
Morphology	**Cubes**	**Truncated Rhombic Dodecahedra**		
		**Surface (110)**	**Surface (100)**	**Bulk**
a (Å)	3.92741(2)	3.91564(2)	3.91151(5)	3.90669(1)
V ($Å^3$)	60.5784(6)	60.0355(5)	59.8458(23)	59.6248(2)
Uiso Sr ($Å^2$)	0.0126(1)	0.0054(1)	0.0132(5)	0.0066(1)
Uiso Ti ($Å^2$)	0.0128(2)	0.0070(2)	0.0126(8)	0.0039(1)
Uiso O ($Å^2$)	0.0753(3)	0.0093(3)	0.0088(13)	0.0080(1)
Weight percentage	-	21.7(2)	7.4(3)	70.8(2)
Equatorial μ strain (Δ*d*/*d*)	0.00368	0.00252	0.00305	0.00182 (w/ 156 nm)
Axial μ strain (Δ*d*/*d*)	0.00324	0.00311	0.00335	
μ strain unique axis (hkl)	100	110	100	isotropic
R(F^2)	4.7	4.1	2.3	3.3
wRp	3.7	2.0		
Zero shift (°)	0.00001	0.00036		
Unique reflections	44	273		
2θ (°)	6.0 to 53.0	6.0 to 74.3		
d-spacing resolution (Å)	0.637	0.467		

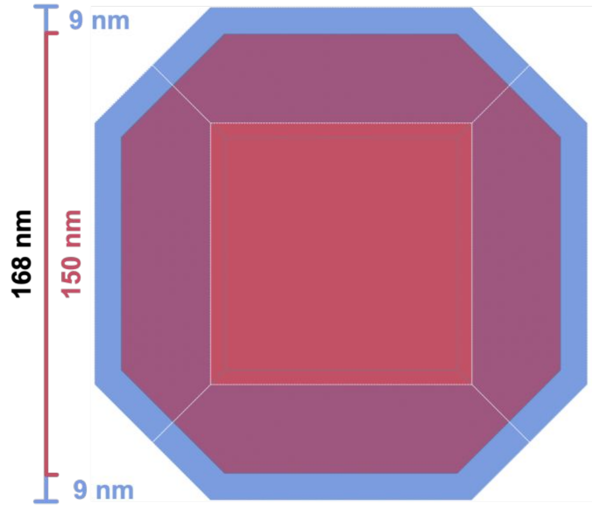

Figure S3. The surface depth ratio of truncated rhombic dodecahedra according to weight percentage of structure refinement. Weight percentages are approximately 29% for the surface layer and 71% for the bulk, relative to the total particle volume.

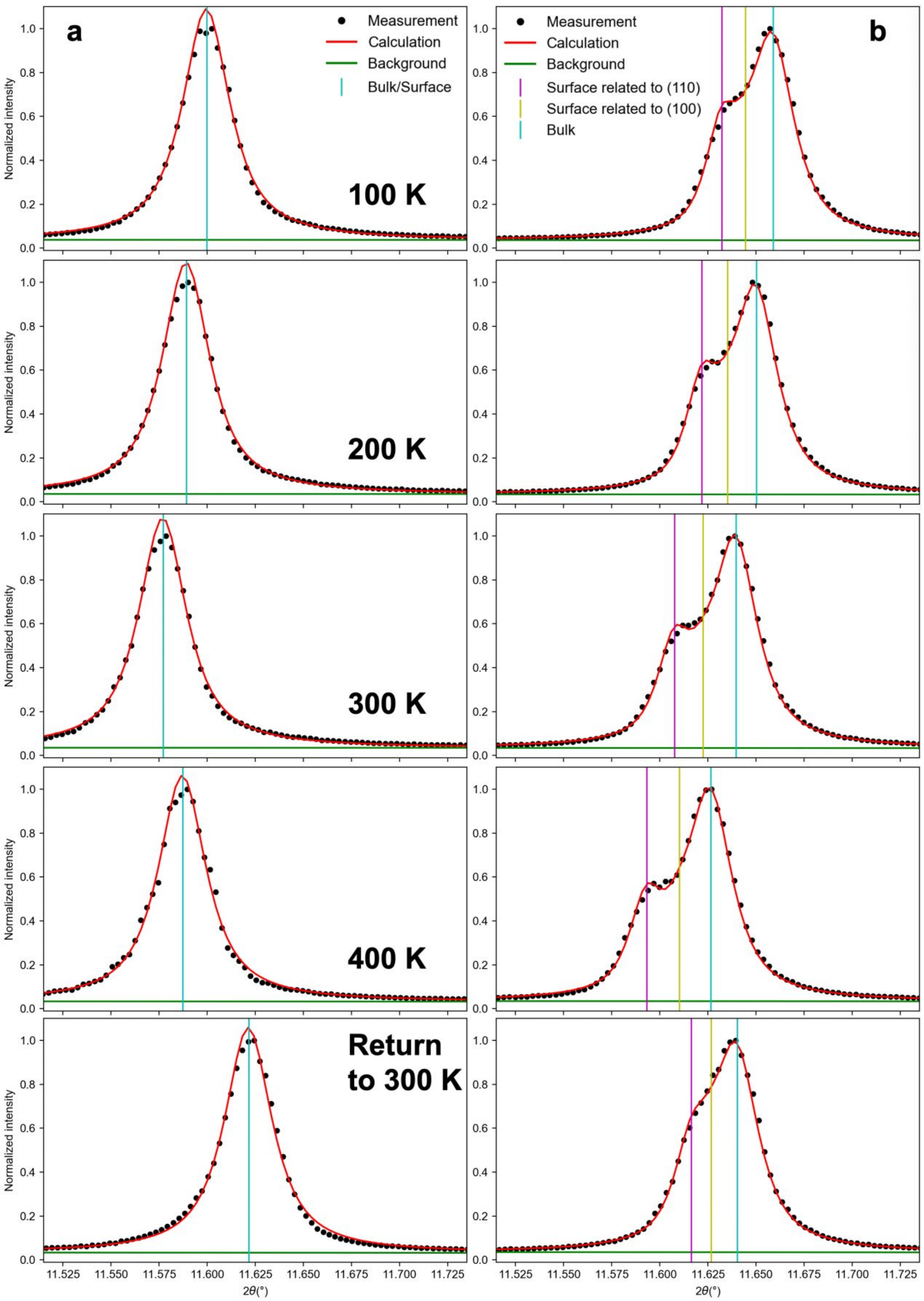

Figure S4. (110) peak shifts of $SrTiO_3$ (a) cubes and (b) truncated rhombic dodecahedra as a function of the heating temperature and after returning to room temperature.
